# A New Birthweight Reference by Gestational Age: A Population Study Based on the Generalized Additive Model for Location, Scale, and Shape Method

**DOI:** 10.3389/fped.2022.810203

**Published:** 2022-03-21

**Authors:** Qiong Wu, Hui-Yun Zhang, Li Zhang, Yue-Qin Xu, Jin Sun, Nan-Nan Gao, Xiu-Yun Qiao, Yan Li

**Affiliations:** ^1^Department of Pediatrics, Child Health Care Center, The First Affiliated Hospital of Shandong, First Medical University, Jinan, China; ^2^Department of Pediatrics, Shandong Maternal and Child Health Hospital, Jinan, China

**Keywords:** child public health, growth chart, birthweight, early growth, gestational age

## Abstract

**Background:**

It is important to choose a suitable birthweight reference to assess newborns, especially those that are small for gestational age (SGA). Currently, there is no regional standard reference for the north of China or for Shandong province.

**Methods:**

A total of 130,911 data records of singleton, live neonates born at 24–42 weeks of gestation were collected from 2016 to 2018 in Shandong province. A new birthweight-for-gestational age percentile reference was constructed based on the Generalized Additive Model for Location, Scale and Shape (GAMLSS) package in R version 3.5. The established gestational age weight curve was compared separately with the Fenton curve, INTERGROWTH−21st curve, and the Chinese Neonatal Network Standard curve of 2015.

**Results:**

We established the reference values of birthweight by gestational age at the 3rd, 10th, 25th, 50th, 75th, 90th, and 97th percentiles. Newborns had much heavier birthweights than those in the INTERGROWTH-21st and Fenton curves at most gestational ages. Although the newborns' birthweight references were closer to the Chinese Neonatal Network Standard except a few for gestational age, this study and INTERGROWTH-21st had similar birthweight curve shapes.

**Conclusions:**

There are obvious differences among the criteria for newborn birthweights. Therefore, it is more accurate to assess newborns using the local birthweight reference.

## Introduction

As a traditional index, birthweight has been used to evaluate intrauterine fetal growth and nutritional status (1). Small for gestational age (SGA), which are newborns whose birthweight falls below the 10th percentile of the reference population, has been identified as the strongest predictor of neonatal morbidity and mortality by pediatricians (2). Many countries have built their own birthweight standard. Current studies on birthweight standards have focused primarily on developed countries (1, 3–11) with limited research from developing and less developed countries (12–15). In 2008, the International Fetal and Newborn Growth Consortium for the Twenty-first Century (INTERGROWTH-21st) (16) developed guidelines for fetal growth and newborn size. The Fenton growth chart for preterm newborns, a meta-analysis based on six related studies, was updated in 2013 and has been widely used in the United States, Britain, Australia, and many other countries to evaluate the intrauterine growth of newborns (17, 18).

In 1986, the birthweight of newborns with gestational ages from 28 to 44 weeks from 15 cities of China was collected and analyzed. Then, the first Chinese newborns' birthweight percentile reference curve was drawn (19). However, the curve did not distinguish between genders and the method used to analyze the data was relatively simple. In 2015, the Chinese Neonatal Network established the newest nationwide neonatal birthweight reference curve with Generalized Additive Model for Location, Scale, and Shape (GAMLSS) method, which has been used in China (20). Subsequently, other provinces in China also successively carried out relevant research (21, 22).

Birthweight can be affected by factors such as ethnicity (23), socioeconomic status, living conditions and natural environment (24, 25), the level of maternal nutrition, and many other factors (26–28). However, limited by sample choice and study design, there is yet to be a consensus on which reference should be adopted for clinical work. Large differences in socioeconomic status, living conditions, and natural environment between the north and south of China make it inappropriate to use the same birthweight reference. It is necessary to establish different birthweight references for different areas. Shandong Province is in the north of China, with a population of 100 million and annual births more than 1.3 million. Therefore, it is essential to establish a local standard for Shandong province. In our study, we aimed to produce a standard growth curve of gestational-age-specific birthweight based on data from the Shandong province and compare the reference from Shandong with international standards.

## Methods

### Study Design and Participants

From each city in the Shandong province of China one hospital was randomly selected from the secondary and tertiary public hospitals to participate in this research. For cities with a resident population of more than eight million, two hospitals were randomly selected. A total of 12 cities and 17 hospitals were included in the study. The data on live-born newborns admitted to the selected hospitals were collected from September 1, 2016, to August 31, 2018, and newborns born at 24–42 weeks of gestation were chosen for the study.

The inclusion criteria for newborns in this study were gestational age ≥24 weeks and ≤42 weeks based on the last menstrual period (LMP) or early pregnancy ultrasound examination (e.g., 40 weeks + 0 day −40 weeks + 6 days) and singleton birth. Exclusion criteria were any congenital malformations or syndromes. The flowchart for sampling of study participants grouped by gestational age is shown in Supplementary Figure 1. Eventually, a total of 130,212 newborns with gestational age of 24–42 weeks were included in the birth data and remained in the data analysis.

Ethical approval was obtained from the Medical Ethics Committee of the First Affiliated Hospital of Shandong First Medical University. Informed consent was obtained from the parents of study participants.

### Weight Measurements and Data Collection

The birthweight (kg) was measured by an electronic weighing scale, accurate to 10 g, after the umbilical cord was cut. The newborns were weighed twice before the weight was recorded. The data collected were gestational age, sex, birthweight, parity, and mode of delivery.

### Data Analysis and Construction of Growth Charts

After removing outliers from the data, we constructed the birthweight curves with the Generalized Additive Models for Location, Scale and Shape (GAMLSS) model proposed by Rigby and Stasinopoulos (29). The box-plot (30) method was used in this study to eliminate the interference with the extreme values of curve fitting. We described six parameters and arranged them in order of size by box-plot, followed by calculation of the upper and lower limits, quartiles, median, and outliers. The critical value was set at two.

The upper and lower limits of birthweight at each gestational age were exported and data outside that scope was deleted. Growth curves for the 3rd, 10th, 25th, 50th, 75th, 90th, and 97th percentiles of birthweight were constructed and stratified by sex, with the variables gestational age, birthweight, and gender by using the R software (R version 3.5) GAMLSS package (29, 31). The selection of the GAMLSS model for newborn birthweight stratified by sex can be based on the Akaike information criterion (AIC) (32), the Bayesian information criterion (BIC), or Schwarz Bayesian criterion (SBC) (33). Because of the sample size, we chose the SBC, since it can draw smoother curves with more accurate predictions (Supplementary Table 1). The worm plot (34) and Q–Q plot (35) were selected to detect and fit the residual map of the model.

### Comparisons

The established gestational age birthweight curve was compared separately with the Fenton curve (a meta-analysis based on intrauterine growth curves from several developed countries), INTERGROWTH-21st curve (growth curves based on a multi-ethnic prospective study), and the Chinese Neonatal Network Standard (CNNS) curves of 2015 (growth curves based on Chinese native population prospective study).

## Results

The study participants included 68,962 male (53%) and 61,250 female (47%) newborns (male-to female-ratio 1.13:1).

Table 1 shows the birthweight percentiles (3rd, 10th, 25th, 50th, 75th, 90th, 97th) for newborns by gestational age. All the male newborns were heavier than female newborns at birth except some in the 3rd percentile.

**Table 1:** Birthweight (kg) percentiles by gestational age.

**Gestational age, weeks**	**Male**							**Female**						
	**P3**	**P10**	**P25**	**P50**	**P75**	**P90**	**P97**	**P3**	**P10**	**P25**	**P50**	**P75**	**P90**	**P97**
24	0.542	0.632	0.709	0.780	0.851	0.930	1.023	0.553	0.631	0.695	0.745	0.798	0.873	0.977
25	0.601	0.700	0.789	0.871	0.955	1.046	1.149	0.606	0.695	0.768	0.829	0.893	0.978	1.094
26	0.665	0.776	0.876	0.974	1.072	1.175	1.292	0.665	0.765	0.850	0.923	0.999	1.097	1.224
27	0.737	0.859	0.974	1.087	1.202	1.321	1.452	0.728	0.841	0.940	1.028	1.119	1.230	1.370
28	0.816	0.952	1.082	1.214	1.348	1.484	1.631	0.798	0.926	1.040	1.145	1.253	1.379	1.532
29	0.905	1.056	1.202	1.355	1.511	1.665	1.831	0.874	1.019	1.151	1.275	1.403	1.546	1.714
30	1.006	1.172	1.336	1.511	1.69	1.865	2.051	0.958	1.122	1.274	1.421	1.572	1.733	1.917
31	1.120	1.304	1.487	1.684	1.886	2.083	2.291	1.055	1.240	1.412	1.585	1.760	1.942	2.143
32	1.251	1.455	1.658	1.875	2.098	2.315	2.544	1.170	1.375	1.569	1.768	1.969	2.173	2.393
33	1.404	1.628	1.849	2.084	2.324	2.559	2.807	1.308	1.533	1.748	1.972	2.198	2.425	2.667
34	1.580	1.825	2.063	2.313	2.567	2.815	3.079	1.477	1.717	1.951	2.196	2.446	2.694	2.960
35	1.786	2.046	2.297	2.560	2.824	3.083	3.357	1.680	1.931	2.178	2.439	2.706	2.973	3.261
36	2.027	2.291	2.548	2.819	3.093	3.359	3.639	1.918	2.173	2.425	2.694	2.973	3.255	3.560
37	2.292	2.551	2.806	3.079	3.356	3.626	3.911	2.182	2.432	2.683	2.952	3.235	3.523	3.839
38	2.550	2.797	3.044	3.311	3.586	3.855	4.138	2.443	2.681	2.921	3.185	3.463	3.745	4.054
39	2.743	2.978	3.217	3.480	3.752	4.015	4.291	2.642	2.865	3.096	3.356	3.630	3.899	4.188
40	2.862	3.091	3.328	3.594	3.869	4.128	4.395	2.771	2.989	3.216	3.477	3.750	4.012	4.286
41	2.967	3.192	3.427	3.697	3.974	4.229	4.486	2.885	3.099	3.323	3.580	3.848	4.103	4.367
42	3.061	3.285	3.521	3.795	4.075	4.327	4.576	2.987	3.198	3.420	3.675	3.941	4.189	4.443

*P, percentile*.

**Figure 1:**
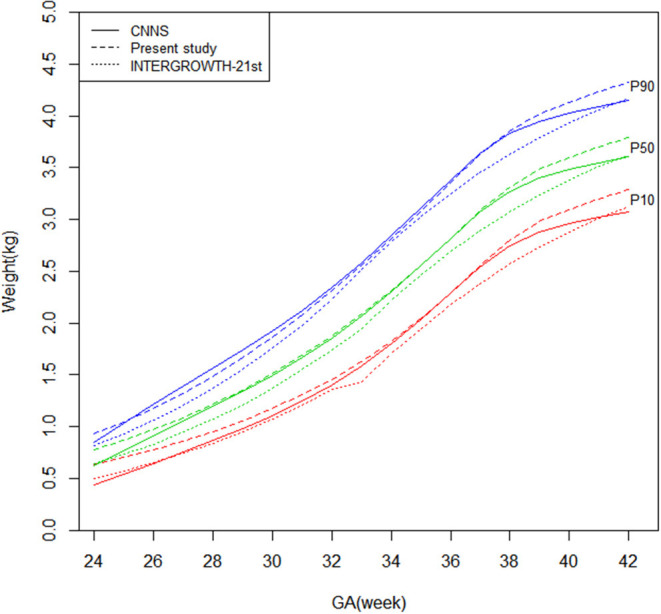
Comparison of birthweight (kg) curves by gestational age among the present study, CNNS and INTERGROWTH-21st Newborns Size Standards/ References (male).

**Figure 2:**
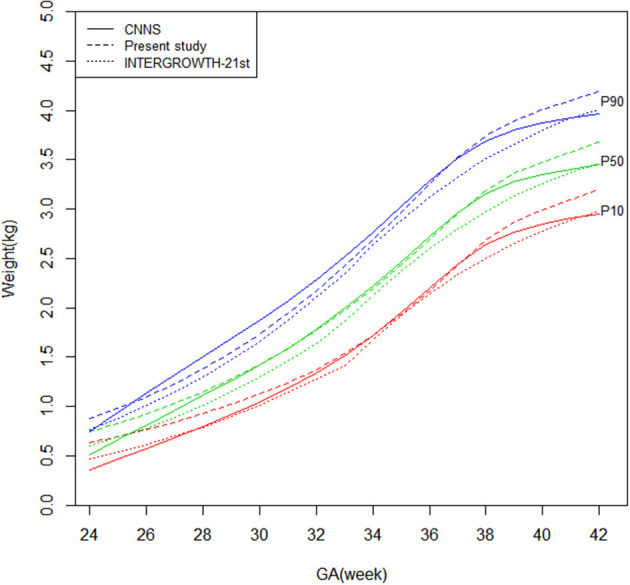
Comparison of birthweight (kg) curves by gestational age among the present study, CNNS and INTERGROWTH-21st Newborns Size Standards/ References (female).

Figures 1, 2 shows newborn birthweight at the 10th, 50th, and 90th percentiles by gestational age based on CNNS and INTERGROWTH-21st. The birthweight curves show similar shapes, although some differences exist for both sexes. As shown, birthweight increased faster in CNNS before 37 weeks of gestation, then flattened out. In the 10th and 50th percentiles, newborns with gestational age from 28–37 weeks had similar birthweights compared to the CNNS curve, but after 37 weeks birthweights gradually increased in our study. In the 10th and 50th percentiles, before 28 weeks of gestation, newborn birthweights in CNNS gradually decreased with decreasing gestational age. In contrast to CNNS, the present study and INTERGROWTH-21st have similar birthweight curve shapes, with slow weight gain before 28 weeks of gestation and a good rate or weight gain after 37 weeks of gestation. However, in the present study, boys were much heavier than in INTERGROWTH-21st.

**Figure 3:**
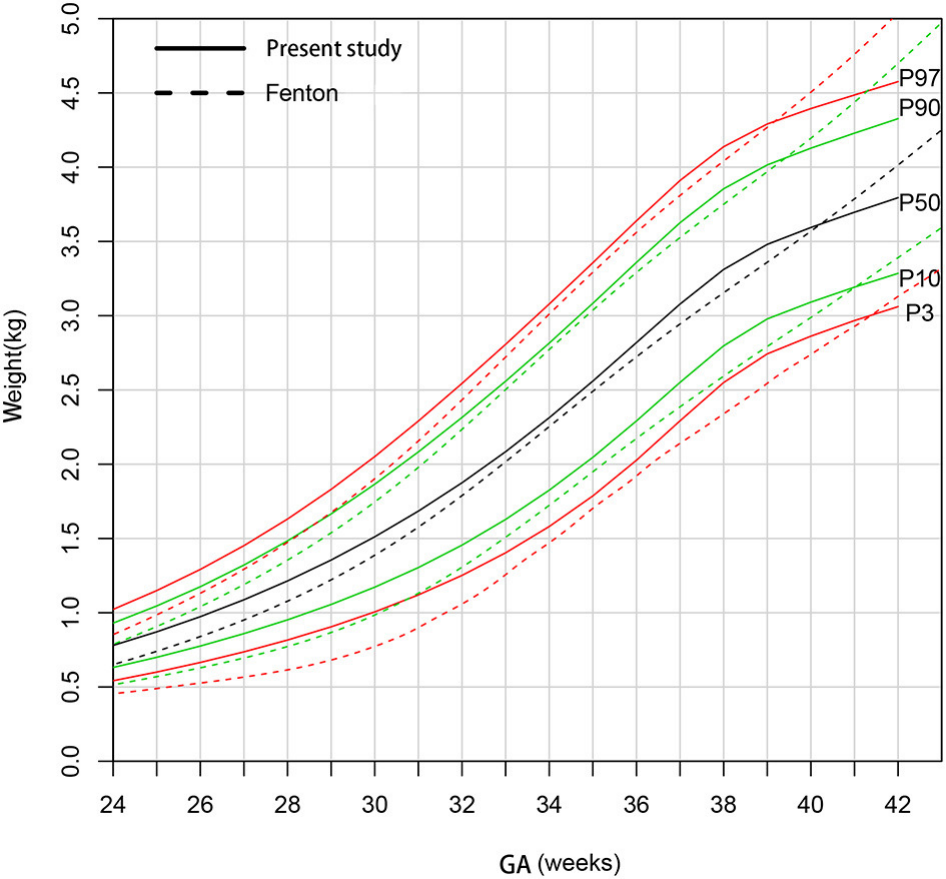
Comparison of birthweight (kg) curves by gestational age between the present study and Fenton curve (male).

Figure 3 shows that the male newborns' birthweight curves in present study are higher than Fenton curves before 39 weeks gestational age and gradually be exceeded after that.

**Figure 4:**
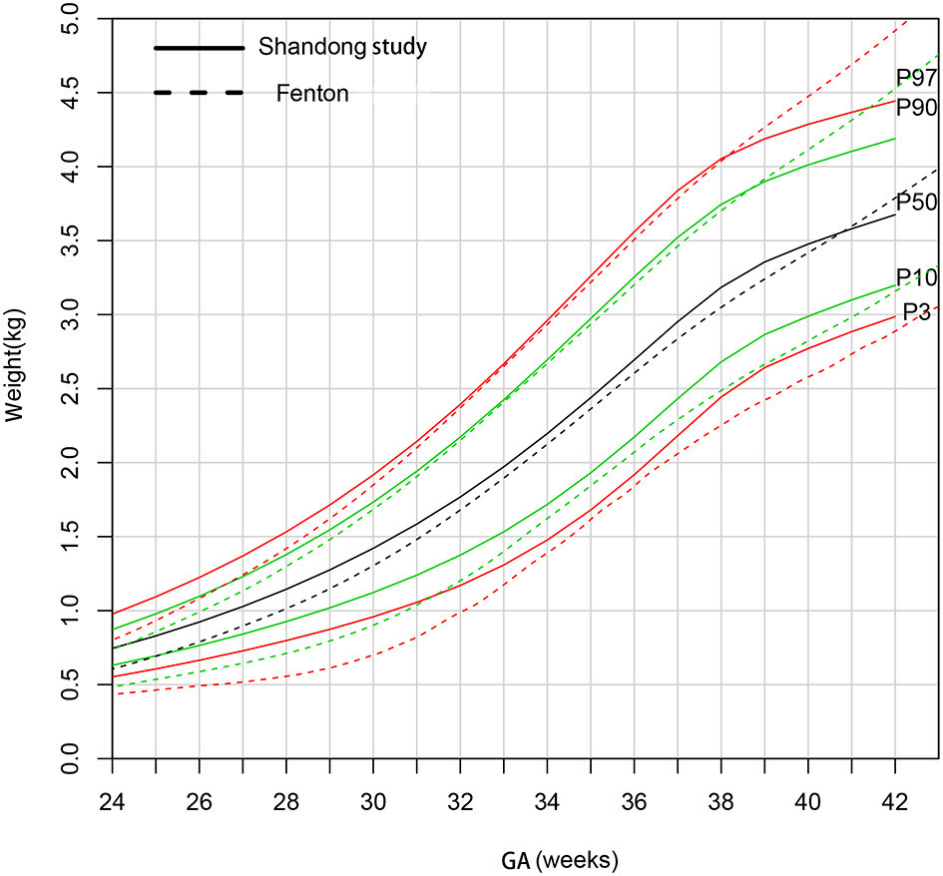
Comparison of birthweight (kg) curves by gestational age between the present study and Fenton curve (female).

Figure 4 shows that the 90th and 97th birthweight curves of female newborns were consistent from 31 to 38 weeks gestational age compared to the Fenton curves. The 50th curve was higher than Fenton curves before 40 weeks gestational age. The 3rd and 10th curves were much heavier than Fenton curves.

## Discussion

It is essential to choose an effective birthweight reference curve to estimate birth outcomes in clinical practice (36–38). Using an outdated standard to screen high-risk neonates may lead to a classification error and thereby mislead the doctors who must decide on clinical diagnosis, treatment, and health resource allocation. Shandong and the other provinces in north China have not yet formulated their local standards. Although the population sample of this study is from 12 cities in Shandong Province, most of the northern China provinces, specifically Liaoning, Jilin, Hebei, and Henan, are mainly Han population and have similar local economic conditions and population migration background. Therefore, in addition to representing Shandong, these data can also represent northern China.

After the Two-Child Policy was implemented in China, many couples tried to have a second baby later in life. This was associated with a greater number of older mothers and assisted fertility methods, both of which increase the risk of low birthweight premature babies (39–41). Premature infants generally have a different pattern of early growth than term infants (42). Assessing these babies properly will improve their prognosis. In China, newborns with gestational age of ≤37 weeks are defined as premature infants.

There are three methods commonly used to construct child growth reference curves: cubic splint function (43); locally-weighted regression and smoothing scatterplots; and coefficient of skewness-median-coefficient of variation, LMS (44). In recent years, LMS, a relatively established method, was widely used in calculating age-related growth references for children and adolescents, such as height, weight, head circumference, and sex development. GAMLSS is an emerging method to construct reference curves for child development. When modeling the variables like gestational age and sex, GAMLSS can use all data in the model; therefore, the distribution curve tends to be stable, even if the sample size is small. In this study, the percentile reference standard of birthweight for Shandong province at a gestational age of 24–42 weeks was created by using the GAMLSS method. The reasons that we chose GAMLSS were the more accurate prediction, smoother curve, and successful use in China and overseas (45, 46). Verified by Q–Q plot, worm plot, and residual plot, our reference standard shows that the data distribution is well-fitted.

Our study corroborates those of the CNNS, showing that newborns in north China are much heavier than those in the Fenton curve and INTERGROWTH-21st. This difference has also been shown in other studies, where Chinese newborns were found to be heavier than those in Europe and the United States (42, 47). There are economic and hereditary reasons to be considered concerning this phenomenon. Comparing recent research data on Chinese birthweights and back to 1986 (19), we found that with improvement in economic levels, the birthweights of term infants were significantly higher than 30 years ago. In addition, pregnant women in China have improved their nutrition during pregnancy, which results in increased weight gain during pregnancy and a heavier baby. On the other hand, gaining too much weight during pregnancy has an adverse effect on blood glucose, which will severely affect birthweight (48–51). The newborns in our study >40 weeks of gestation become much lighter than those in the Fenton reference because our curves were based on intrauterine growth data, while the Fenton reference was based on extrauterine growth data.

In the 10th percentile, most of the gestational ages show much heavier birthweights in our study than those in the CNNS, which might result from genetic, economic, and geographic factors. Most importantly, there will be a more accurate assessment for SGA newborns in north China if we use the birthweight reference from Shandong.

Our research has several limitations. First, because of the different medical treatment levels in different regions, higher birthweights are associated with higher chances of survival. Our data comes from level II or level III public hospitals and the medical treatment level at these hospitals is relatively high. Although we can collect more data on SGA newborns, this can cause sampling error, so that birthweight in our study is slightly high, especially for newborns with gestational age <28 weeks. Second, our study is a cross-sectional study, and more follow-up is needed to observe weight fluctuations. In future studies, we can establish an array of research including local newborns with larger sample size, complete sets of growth measurements like birth height and birth head circumference, and long-term follow-up, and construct a more reliable growth curve for newborns especially for newborns with gestational age <28 weeks.

## Conclusions

It is important to choose suitable criteria to assess newborn birthweight. We established the first birthweight references from Shandong Province. Our birthweight references are higher than those of Fenton and INTERGROWTH-21st and are somewhat higher than those of the CNNS. Although the reason for this needs to be further clarified, it might indicate possible economic and hereditary differences and creates concern over the appropriateness of Fenton, INTERGROWTH−21st, and the CNNS in assessing the local newborn population. Therefore, it is necessary to construct and use regional birthweight standards for newborns from northern China.

## Data Availability Statement

The original contributions presented in the study are included in the article/Supplementary Material, further inquiries can be directed to the corresponding author.

## Author Contributions

YL and QW designed the study. YL and LZ revised the manuscript. QW and H-YZ constructed growth charts and wrote the manuscript. LZ, Y-QX, and JS data collection and organization. N-NG and X-YQ data analyses. YL had primary responsibility for final content. All the authors guided and give many useful suggestion for this research, they both read and approved the final manuscript.

## Conflict of Interest

The authors declare that the research was conducted in the absence of any commercial or financial relationships that could be construed as a potential conflict of interest.

## Publisher's Note

All claims expressed in this article are solely those of the authors and do not necessarily represent those of their affiliated organizations, or those of the publisher, the editors and the reviewers. Any product that may be evaluated in this article, or claim that may be made by its manufacturer, is not guaranteed or endorsed by the publisher.
