# Supplementary material for: A New Birthweight Reference by Gestational Age: A Population Study Based on the Generalized Additive Model for Location, Scale, and Shape Method
Source: Front Pediatr. 2022 Mar 21;10:810203. doi: 10.3389/fped.2022.810203 (PMC8978627; doi:10.3389/fped.2022.810203)
Supplement: Supplementary file 1 [file Table_1.DOCX]

Supplementary Table 1 Selection of GAMLSS model△*.

| Distribution | Deviance | SBC |
| --- | --- | --- |
| BCPE | 137084.6 | 137320.2 |
| BCT | 137161.6 | 137397.1 |
| BCTo | 137035.5 | 137235.7 |
| BCCG | 137249.3 | 137425.9 |
| BCCGo | 137095.2 | 137271.9 |
| BCPEo | 136931.5 | 137167 |

△Abbreviation: BCCGo, Box-Cox Cole-Green orig. ; BCPEo: Box-Cox power exponential distribution orig. ; BCTo: Box-Cox t orig. ; GAMLSS: Generalized Additive Models for Location, Scale and Shape;.

* Model selection was according to the Akaike information criterion (AIC) and the Bayesian information criterion (BIC) or Schwarz Bayesian criterion (SBC).
