# Supplementary figures and images for: A New Birthweight Reference by Gestational Age: A Population Study Based on the Generalized Additive Model for Location, Scale, and Shape Method
Source: Front Pediatr. 2022 Mar 21;10:810203. doi: 10.3389/fped.2022.810203 (PMC8978627; doi:10.3389/fped.2022.810203)

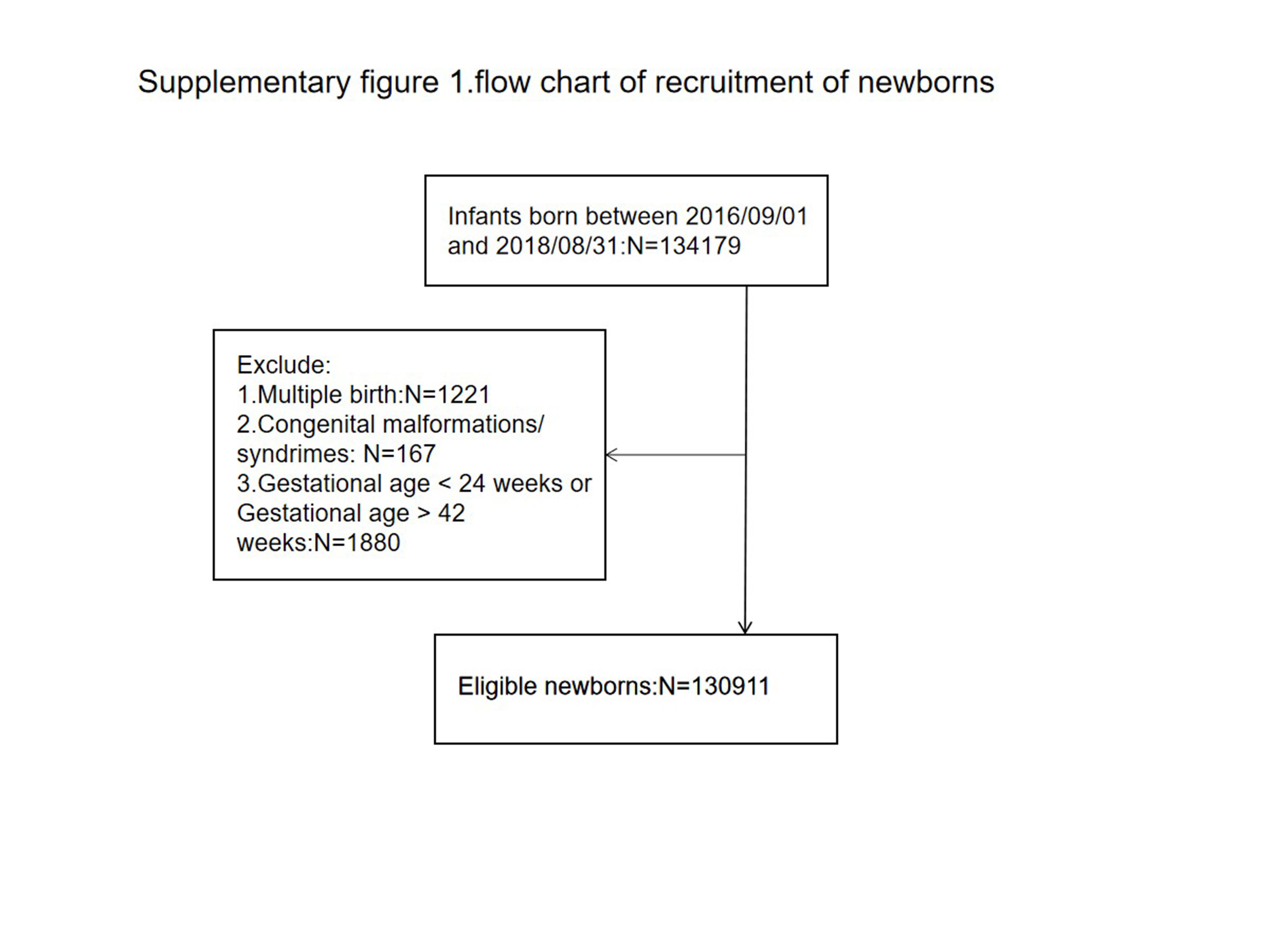

Supplement: Supplementary file 2 [file Image_1.JPG]
